# Pleomorphic bacteria-like structures in human blood represent non-living membrane vesicles and protein particles

**DOI:** 10.1038/s41598-017-10479-8

**Published:** 2017-09-06

**Authors:** Jan Martel, Cheng-Yeu Wu, Pei-Rong Huang, Wei-Yun Cheng, John D. Young

**Affiliations:** 1 Center for Molecular and Clinical Immunology, Chang Gung University, Taoyuan, Taiwan; 2 Laboratory of Nanomaterials, Chang Gung University, Taoyuan, Taiwan; 3 Chang Gung Immunology Consortium, Linkou Chang Gung Memorial Hospital, Taoyuan, Taiwan; 4 Research Center of Bacterial Pathogenesis, Chang Gung University, Taoyuan, Taiwan; 5 Department of Molecular and Cellular Biology, College of Medicine, Chang Gung University, Taoyuan, Taiwan; 6 Laboratory of Cellular Physiology and Immunology, Rockefeller University, New York, NY USA; 7 Biochemical Engineering Research Center, Ming Chi University of Technology, Taipei, Taiwan

## Abstract

Although human blood is believed to be a sterile environment, recent studies suggest that pleomorphic bacteria exist in the blood of healthy humans. These studies have led to the development of “live-blood analysis,” a technique used by alternative medicine practitioners to diagnose various human conditions, including allergies, cancer, cardiovascular disease and septicemia. We show here that bacteria-like vesicles and refringent particles form in healthy human blood observed under dark-field microscopy. These structures gradually increase in number during incubation and show morphologies reminiscent of cells undergoing division. Based on lipid analysis and Western blotting, we show that the bacteria-like entities consist of membrane vesicles containing serum and exosome proteins, including albumin, fetuin-A, apolipoprotein-A1, alkaline phosphatase, TNFR1 and CD63. In contrast, the refringent particles represent protein aggregates that contain several blood proteins. 16S rDNA PCR analysis reveals the presence of bacterial DNA in incubated blood samples but also in negative controls, indicating that the amplified sequences represent contaminants. These results suggest that the bacteria-like vesicles and refringent particles observed in human blood represent non-living membrane vesicles and protein aggregates derived from blood. The phenomena observed during live-blood analysis are therefore consistent with time-dependent decay of cells and body fluids during incubation *ex vivo*.

## Introduction

Live-blood analysis (LBA) is a popular alternative medicine procedure often used in combination with therapies such as chiropractic treatments. During LBA, a drop of blood is observed under a dark-field microscope without fixation or staining. While the blood of healthy humans is usually considered a sterile environment^1^, LBA proponents have claimed that pleomorphic bacteria are found in the blood of healthy and diseased humans^2^. Moreover, it has been claimed that this technique can be used to evaluate immune system status and diagnose several forms of allergies and chronic diseases, including cancer, cardiovascular disease and immunity-related disorders^2^. Yet, LBA has never been examined in detail and its use remains controversial.

A large body of historical literature has reported that human blood is infected by overlooked microorganisms associated with aging, degenerative diseases and cancer (reviewed previously^3–5^). In the early 1900’s, Béchamp claimed that animal body fluids contained subcellular living particles (i.e., *microzymas*) that transformed into bacteria upon death and decay of the host^6^. Enderlein described small entities called *endobionts* and *protits* in human blood and believed that these particles underwent a complex life cycle that correlated with disease progression^7^. Similar observations were made in the 1950’s by Villequez who proposed that human blood was infected by a latent parasite similar to bacterial L-forms (i.e., bacteria lacking a cell wall)^8, 9^. Tedeschi^10–12^ and Pease^13–16^ reported that the blood of healthy and diseased individuals appeared to be continually infected with bacteria. Naessens described small living blood particles, which he called *somatids*, as part of a complex life cycle that may culminate in the formation of pathogenic bacterial forms under disease conditions^4, 17^.

While many of these observations were made before the advent of modern molecular biology analyses, recent studies have provided further support to the possibility that pleomorphic bacteria may exist in human blood. Using dark-field microscopy, transmission electron microscopy (TEM), PCR and flow cytometry, McLaughlin *et al*. described pleomorphic bacteria in the blood of every healthy individuals tested^18^. Nikkari and colleagues performed a PCR analysis of human blood and observed that larger amount of bacterial DNA was found in blood samples obtained from healthy individuals compared to matched PCR reagent controls^19^. Similarly, Potgieter *et al*. suggested that dormant blood bacteria possibly originating from the gut microbiota may play a role in chronic inflammation and the development of various diseases^20^. Nonetheless, the nature of these provoking claims and their possible implications for blood transfusions and disease transmssion remain to be examined.

We have shown earlier that mineralo-organic nanoparticles spontaneously form in human serum when calcium, carbonate, phosphate and other ions exceed saturation^21–38^. The description of these mineral particles has helped us to resolve the controversy surrounding the existence of nanobacteria—small entities claimed earlier to represent the smallest bacteria on earth and the cause of various human diseases^39–41^. Contrary to previous claims, our work has shown that nanobacteria represent non-living mineralo-organic nanoparticles possessing various biomimetic properties, including the formation of bacteria-like morphologies^21–23, 32^, the possibility to grow, proliferate and propagate by subculture^22, 32, 33^, and the ability to bind to organic molecules^22, 27, 37^.

In the present study, we examined the blood of healthy human volunteers under dark-field microscopy and TEM. While our observations show that pleomorphic bacteria-like structures and refringent particles form in human blood, our results indicate that these entities represent non-living membrane vesicles and protein aggregates that mimic live bacteria in various ways. These findings suggest that many aspects of the past literature on the existence of blood endoparasites and the use of LBA should be re-evaluated from an entirely different perspective.

## Results

### Formation of bacteria-like structures in human blood

**Figure 1:**
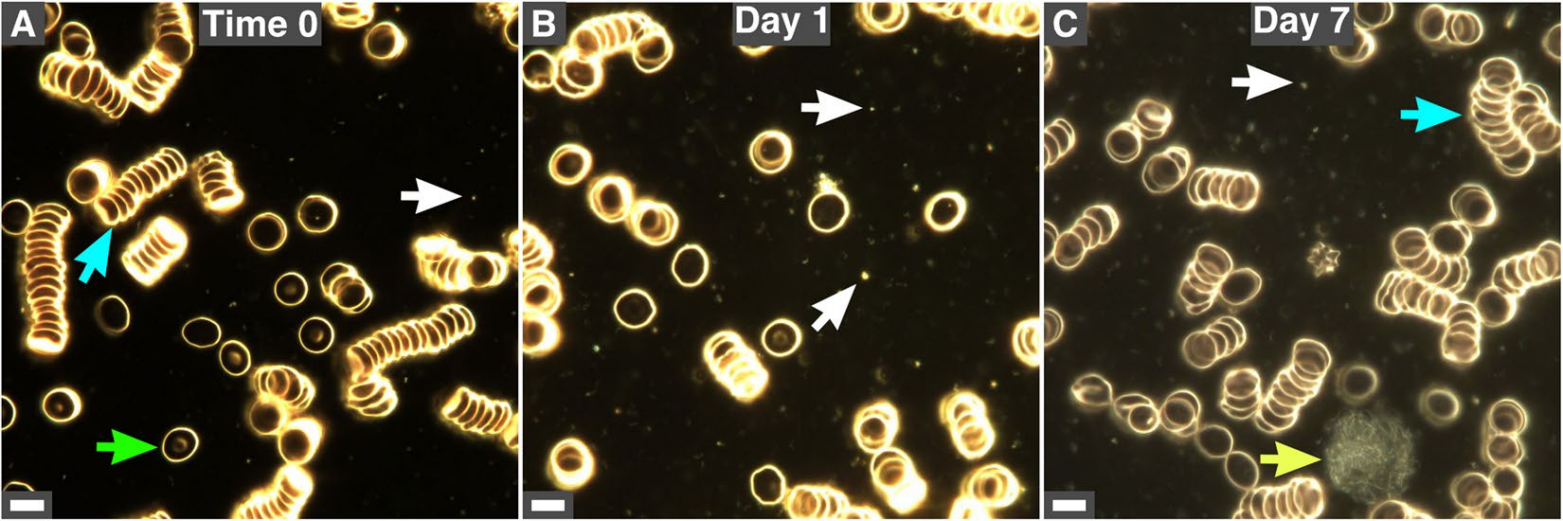
Observation of human blood under dark-field microscopy. (**A**) Healthy human blood was observed under dark-field microscopy without fixation or staining. Samples were observed immediately after blood withdrawal (time 0). Blood samples were incubated at 30 °C for one day (**B**) or one week (**C**). The representative images presented here show RBCs as single cells (green arrow) or aggregates (blue arrows). Refringent particles (white arrows) gradually increased in number with time. Structures representing cellular debris possibly originating from white blood cells were also observed (yellow arrow in **C**). Scale bars: 8 μm.

We examined peripheral blood from 30 healthy human subjects using dark-field microscopy without fixation or staining. Red blood cells (RBCs) were observed as single cells (Fig. 1A, green arrow) or aggregates (Fig. 1A and C, blue arrows). Refringent particles that vibrated randomly were found in all samples (Fig. 1A–C, white arrows). Cellular debris were also observed following incubation (Fig. 1C, day 7, yellow arrow).

**Figure 2:**
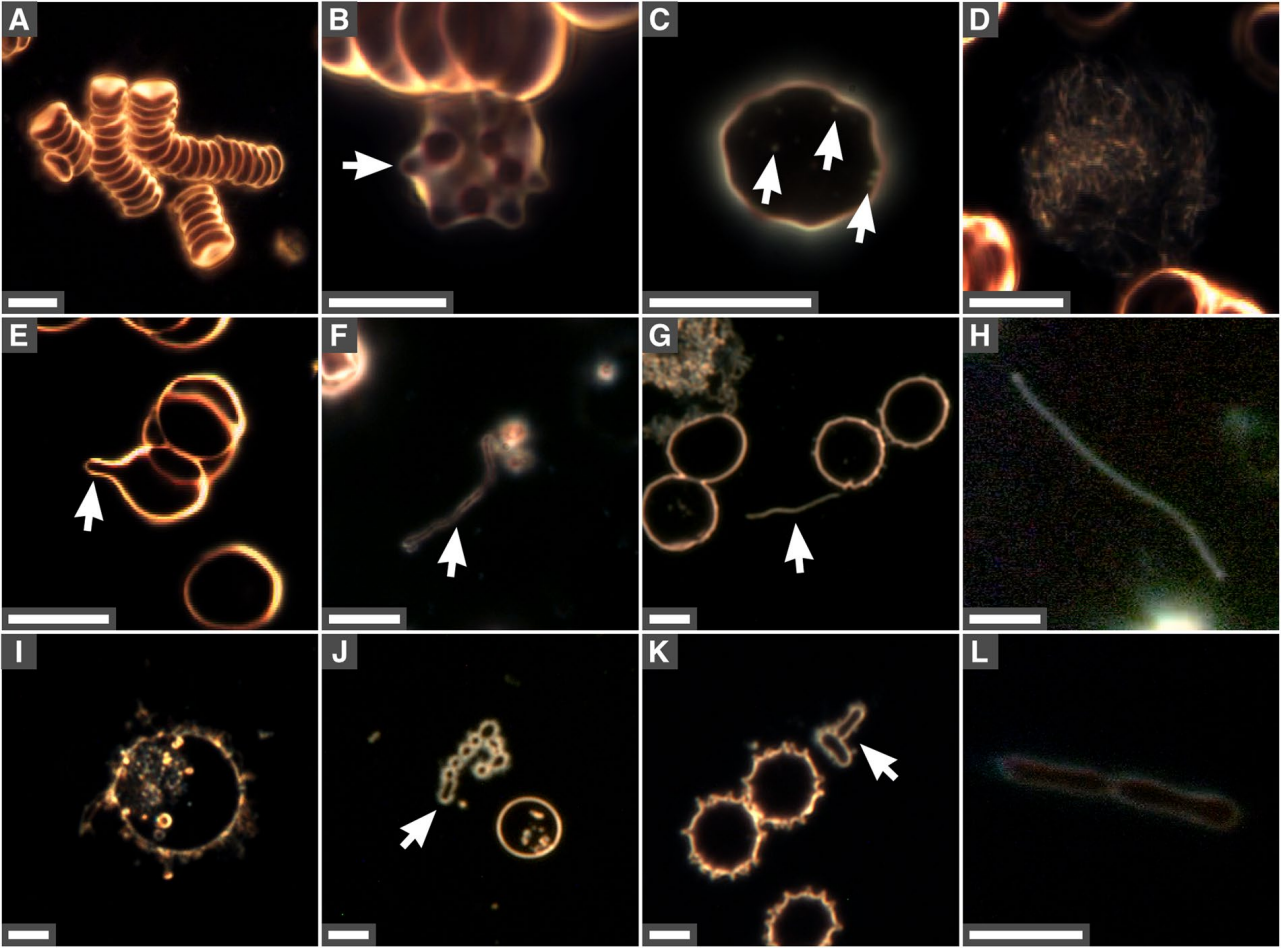
Changes observed in red blood cells and formation of bacteria-like forms in incubated human blood. Aliquots of human blood were incubated at 30 °C for seven days and observed under dark-field microscopy without fixation or staining. (**A**) Aggregated RBCs; (**B**) RBC with spicules (arrow); (**C**) particles inside RBC (arrows); (**D**) filamentous cell debris; (**E**) RBC vesiculation with spicules or tubule formations (arrow); (**F**) membrane structures; (**G,H**) elongated bacteria-like forms (arrow in **G)**; (**I**) intracellular vesicles; (**J**) coccoid-like vesicles (arrow); (**K**) bacteria-like forms (arrow); (**L**) bacteria-like structures similar to cells undergoing division. Scale bars: 8 μm.

In addition to aggregated blood cells (Figs 1A–C and 2A), RBCs with membrane projections were observed in incubated blood (Figs 2B, 7-day incubation). Some RBCs contained intracellular particles (Fig. 2C) similar to the ones observed around cells (Fig. 1A–C). Cellular debris were also noticed in incubated blood (Fig. 2D, enlarged from Fig. 1C). During prolonged incubation, spicules or tubule formations protruded from RBCs (Fig. 2E, arrow). Some of these projections extended further and eventually detached from RBCs, forming elongated filaments (Fig. 2F–H). In addition, small pleomorphic membrane-delineated vesicles were observed, both inside and outside RBCs (Fig. 2I–L), with some vesicles forming structures resembling bacteria undergoing cell division (Fig. 2J and L).

**Figure 3:**
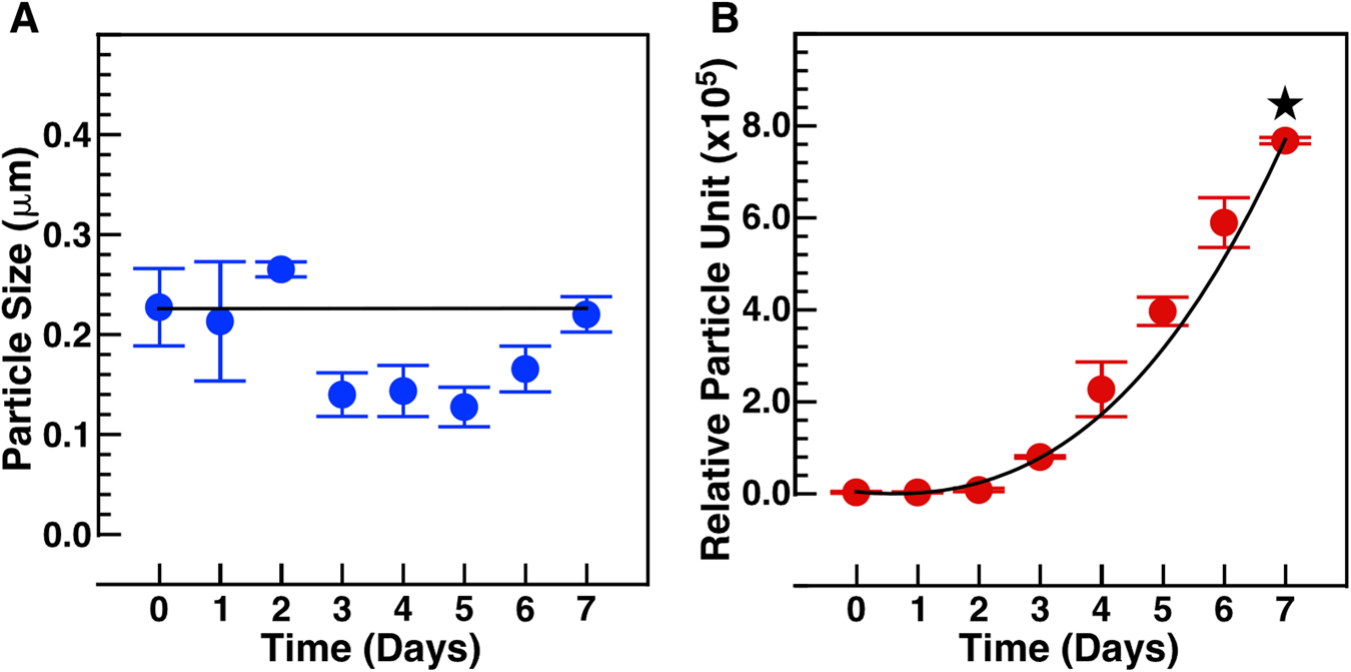
Bacteria-like forms and blood particles increase in number during incubation. Fresh human blood (time 0) or human blood incubated for 1 to 7 days was centrifuged at 800 × *g*, followed by filtration through 0.2 μm filter to remove cells. Particle size (**A**) and number (**B**) was determined using DLS as described in *Methods*. The star symbol denotes *p* < 0.01 vs. time 0. The data represent means ± standard deviation of three independent experiments.

To verify whether the bacteria-like forms proliferate, we incubated human blood at 30 °C for several days and determined the size distribution and number of bacteria-like forms and refringent particles during incubation using dynamic light scattering (DLS) (Fig. 3A and B). While the size of the particles remained relatively constant throughout the period of observation (Fig. 3A, ~230 nm), the number of vesicles and particles increased in a time-dependent manner, reaching relatively high numbers after 6 and 7 days (Fig. 3B). On the other hand, inoculation of incubated blood aliquots onto blood agar and brain heart infusion agar remained sterile (data not shown), indicating that no bacterial contamination had occurred.

### Electron microscopy observations of pleomorphic bacteria-like in human blood

**Figure 4:**
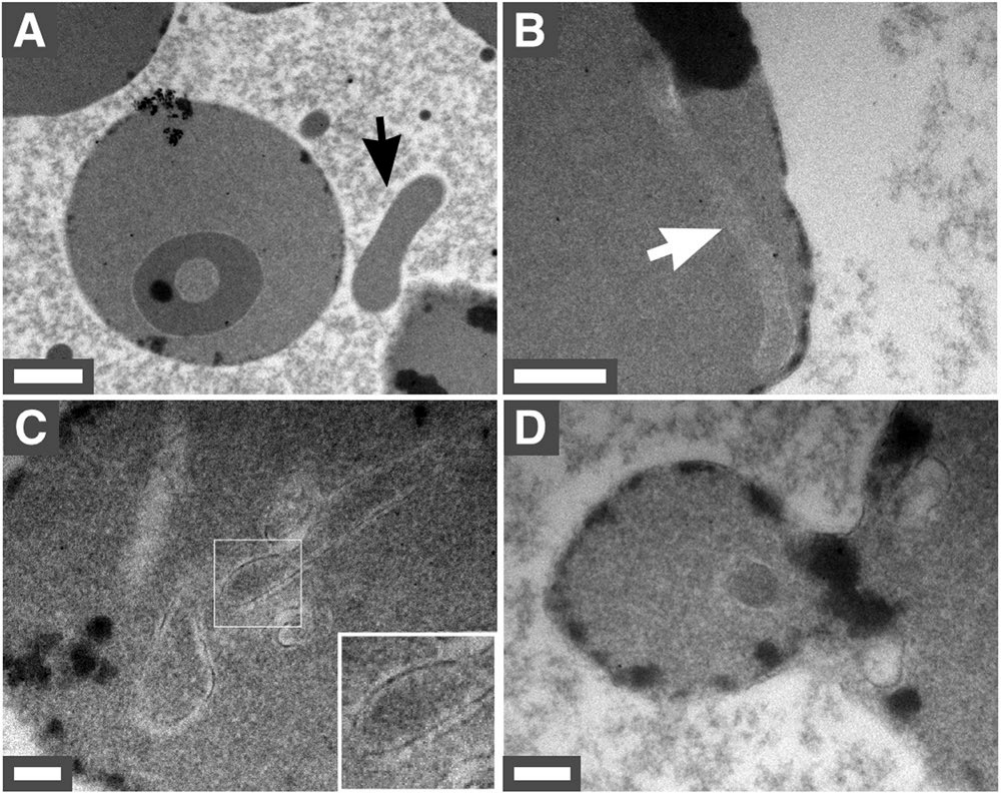
Pleomorphic bacteria-like forms observed under thin-section TEM. Human blood incubated for seven days was processed for TEM as described in *Methods*. (**A**,**B**) Bacteria-like forms were found both inside and outside RBCs (arrows). (**C**) Bacteria-like forms appeared to be delineated by a lipid membrane (inset). (**D**) Formation of a membrane vesicle by RBC vesiculation. Scale bars: 2 μm.

To examine the morphology and internal organization of the pleomorphic bacteria-like forms observed in blood, we processed samples of incubated blood for thin-section TEM. Figure 4A shows a representative image of a RBC along with a small bacteria-like form (denoted by an arrow). Bacteria-like forms were also noted within RBCs (Fig. 4B and C, arrows). The structures were delineated by a cellular membrane (Fig. 4C, inset) and seemed to be devoid of organelles or bacterial cell wall. In addition, TEM images revealed RBCs undergoing vesiculation in incubated blood (Fig. 4D). These findings are consistent with the dark-field microscopy observations shown above (Figs 1 and 2).

### Biochemical composition of bacteria-like forms isolated from blood

**Figure 5:**
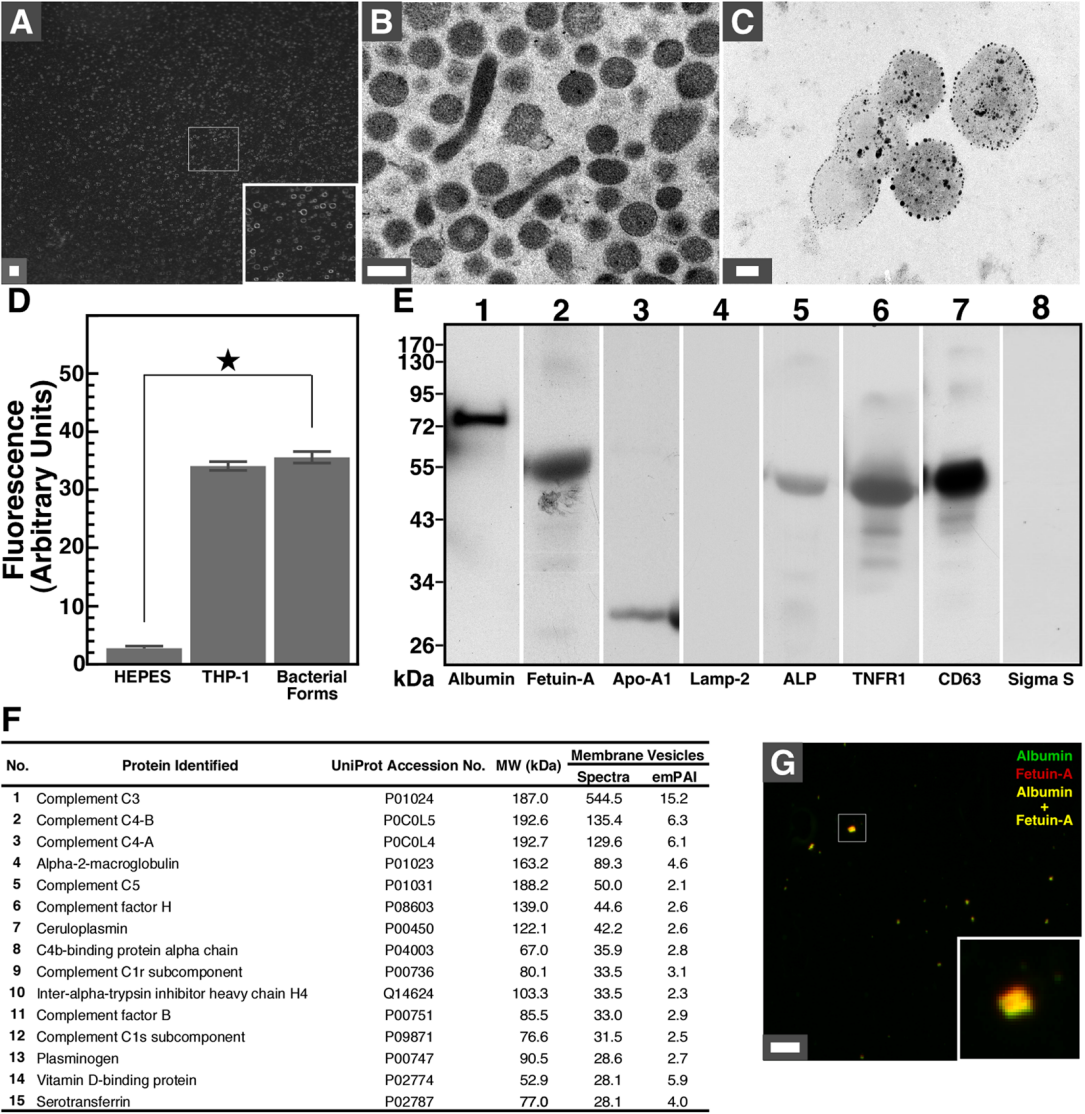
Biochemical characterization of bacteria-like forms isolated from human blood. Human blood was incubated for seven days and bacteria-like forms were isolated by centrifugation, before processing for (**A**) dark-field microscopy, (**B**) thin-section TEM, (**C**) annexin V staining, (**D**) DiR staining, (**E**) Western blotting, (**F**) proteomics analysis and (**G**) immunofluorescence as described in *Methods*. In (**A**), the isolated bacteria-like forms show homogenous composition (see inset). In (**B**), the isolated vesicles show round and bacillus-like forms delineated by a membrane. In (**C**), bacteria-like forms observed under TEM were stained with annexin V conjugated with gold nanoparticles. In (**D**), bacteria-like forms were stained with DiR as did THP-1 cells used as positive control while HEPES buffer used as a negative control showed no reaction. The star symbol denotes *p* < 0.01 vs. control HEPES. In (**E**), proteins isolated from bacteria-like forms consist of common blood cell proteins commonly found in blood and exosomes. Human lysosomal protein Lamp-2 or bacterial RNA polymerase sigma S factor was not detected. Cropped Western blots showing full lanes are shown here; full, uncropped blots are provided in Supplementary Figure 1. In (**F**), the bacteria-like structures contain various blood proteins (for a complete list, see Supplementary Table 1). Proteins were ranked by the number of matching spectra. Exponentially modified protein abundance index (emPAI) values are also shown. In (**G**), yellow protein particles containing albumin and fetuin-A are shown (inset). Scale bars: 8 μm (**A**); 200 nm (**B**,**C**); (**G**) 500 nm.

To study the nature of bacteria-like forms, we processed aliquots of incubated blood through a 0.2-μm pore membrane and isolated a fraction of bacteria-like structures showing round coccoid shapes (Fig. 5A, inset). Under TEM, round vesicles with a diameter of ~200 nm were observed, along with elongated 600-nm-long filamentous forms (Fig. 5B). Bacteria-like forms were delineated by a membrane and contained granular material (Fig. 5B). Bacteria-like forms were stained with annexin-V-coupled gold nanoparticles (Fig. 5C), indicating that phosphatidylserine was exposed on the surface of the vesicles. The bacteria-like entities were also stained with the lipid tracer DiR (Fig. 5D), confirming that they were delineated by a lipid membrane. A positive control consisting of human THP-1 macrophages also stained with DiR, whereas the HEPES buffer used as a negative control showed no reaction (Fig. 5D).

We also examined the protein composition of the vesicles by performing Western blotting using a series of antibodies that react against serum proteins (Fig. 5E). Our results showed that bacteria-like forms contained blood proteins such as human serum albumin (HSA), human serum fetuin-A (HSF) and apolipoprotein-A1 (Apo-A1) (Fig. 5E). The vesicles also harbored the membrane protein alkaline phosphatase (ALP; Fig. 5E) which is commonly found on the surface of blood cells^42^. In addition, the vesicles contained tumor necrosis factor receptor 1 (TNFR1) and cluster of differentiation 63 (CD63) (Fig. 5E), two ubiquitous cell-surface proteins found respectively in exosome-like vesicles and exosomes^43^. On the other hand, neither lysosomal-associated membrane glycoprotein-2 (Lamp-2), a protein found in lysosomes and on the surface of activated leukocytes, nor RNA polymerase sigma factor S (sigma S), a protein commonly found in bacteria, was detected (Fig. 5E).

To identify the proteins found in the vesicles, we performed a proteomics analysis of isolated vesicles (see Fig. 5F for an abbreviated list of the proteins identified; the full list is shown in Supplementary Table 1). We used a comprehensive proteomics analysis developed earlier to study the proteins that bind to mineralo-organic nanoparticles^27^. A wide range of blood proteins was found in blood vesicles, including HSA, HSF and Apo-A1 (Supplementary Table 1). Several other blood proteins were detected in blood particles, including complement proteins, protease inhibitors and lipid carriers (Supplementary Table 1). On the other hand, search against protein databases of *Escherichia coli* or other bacteria failed to identify any proteins (data not shown), indicating that the proteins found in the vesicles were not of bacterial origin. Notably, co-localization of HSA and HSF within blood-derived vesicles was confirmed by immunofluorescence (Fig. 5G).

Analysis of the lipid composition of the vesicles revealed the presence of phospholipids and cholesterol (Table 1), supporting our observations that the vesicles contain a cell membrane similar to that of eukaryotic cells (Fig. 1B, inset, Fig. 2J–L). Lipid analysis revealed a minimal amount of triglycerides (Table 1), suggesting that the vesicles were not lipoproteins. Based on these results, we conclude that the bacteria-like forms represent membrane vesicles derived from blood cells.

**Table 1:** Lipid composition of pleomorphic bacteria-like forms isolated from human blood.

Lipid	Concentration (μM)
Phospholipid	56.48 ± 14.72
Cholesterol	21.33 ± 2.08
Triglyceride	0.06 ± 0.01

Lipid analysis was performed using commercial detection kits as described in *Methods*. The data represent means ± standard deviation of three independent experiments.

### Refringent particles in blood represent protein aggregates

**Figure 6:**
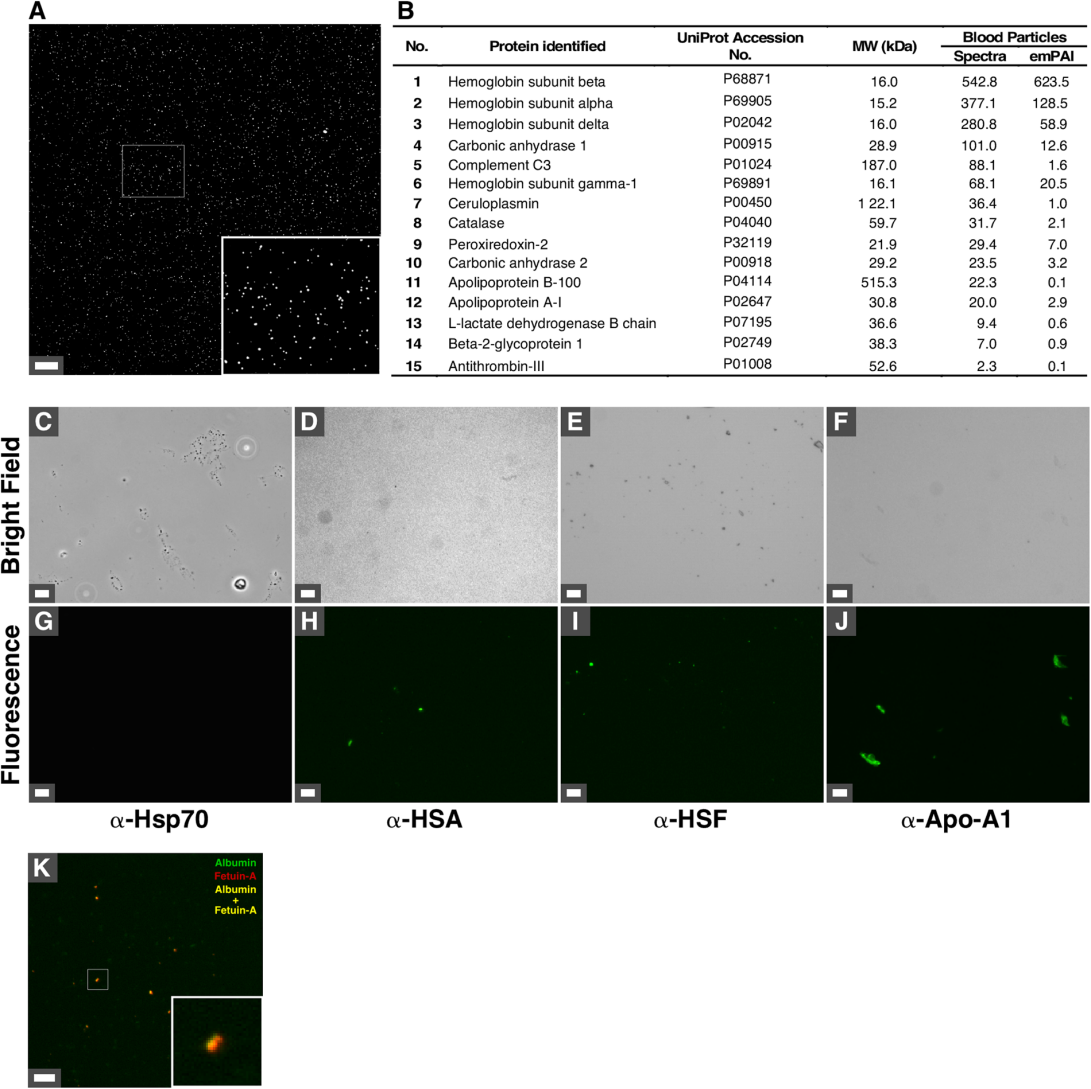
Biochemical characterization of refringent particles isolated from human blood. Human blood was incubated for seven days at 30 °C, before centrifugation to remove blood cells and treatment with Triton X-100 to remove membrane-bound vesicles as described in *Methods*. (**A**) The blood particles obtained this way were observed by dark-field microscopy to confirm homogeneity (see inset). (**B**) Blood particles were submitted to proteomics analysis (see *Methods*) and a large number of blood proteins were identified (see Supplementary Table 2 for a complete list). Proteins were ranked by the number of matching spectra. emPAI values are also shown. Specimens of blood particles were observed under bright field optical microscopy (**C**–**F**) and immunofluorescence (**G**–**J**) using the indicated antibodies (anti-human serum albumin, α-HSA; anti-human serum fetuin-A, α-HSF; anti-apolipoprotein-A1, α-Apo-A1). Anti-Hsp 70 antibody (α-Hsp 70) was used as negative control. (**K**) Immunofluorescence revealed the presence of yellow-orange particles containing albumin and fetuin-A (see inset). Scale bars: (**A**, **C**–**J**) 1 μm; (**K**) 500 nm.

In addition to the cell-derived vesicles described above, we examined the composition of the refringent particles observed under dark-field microscopy in incubated human blood (Fig. 1A–C, white arrows). We first treated the isolated bacteria-like forms described in the previous section with the detergent Triton X-100 in order to disrupt cellular membranes. The supernatant of the resulting fraction was transferred onto glass slides and observed under dark-field microscopy. As seen in Fig. 6A, the isolated blood particles were homogenous and similar to the particles observed earlier in incubated blood (Fig. 1A–C, white arrows).

**Figure 7:**
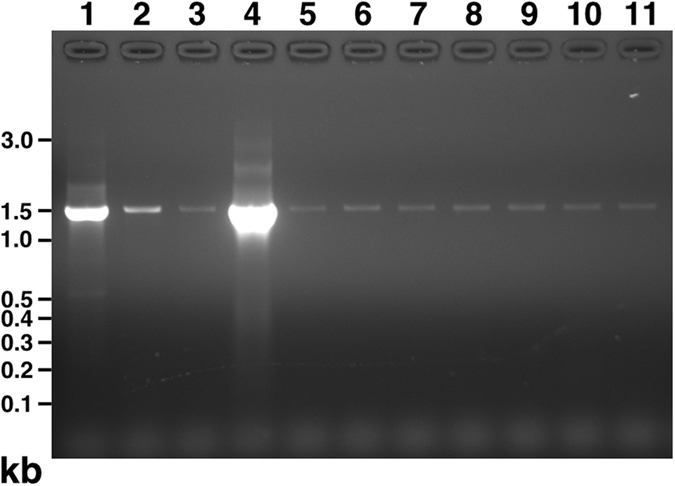
Bacterial 16S rDNA detected in human blood samples. Samples of human blood prior to incubation (lane 9) or following incubation for one day (lane 10) or seven days (lane 11) were filtered through 0.2 μm filter and processed for PCR as described in the *Methods*. PCR products were separated on agarose gel and stained with ethidium bromide. Positive controls consisted of genomic DNA isolated from *E. coli* strain DH5α (lane 1) or Stbl3 (lane 2), *F. nucleatum* (lane 4), or an aliquot of culture medium of DH5α (lane 3) or *F. nucleatum* (lane 5). Double-distilled water processed or not as for DNA extraction (lane 6 and lane 7, respectively) and DNA extraction buffer processed as for extraction (lane 8) were used as negative controls. PCR products of 1.5 kb of high or low intensity were detected in each lane.

We submitted the blood particles to proteomics analysis (see Fig. 6B for an abbreviated list of proteins; the full list is shown in Supplementary Table 2). Various blood proteins were found in the particles, including HSF and Apo-A1 (Supplementary Table 2). Other blood proteins detected in blood particles included complement proteins, protease inhibitors and lipid carriers (Supplementary Table 2). We also identified proteins in samples of particles that had been autoclaved (Supplementary Table 2; “Autoclaved particles”). In this case, the number of proteins and signal intensity was considerably lower than non-autoclaved samples, consistent with protein degradation. Similar to the proteomics results described above for the blood vesicles (Supplementary Table 1), the proteomics data of blood particles was searched against bacterial protein databases but this analysis did not yield any proteins (data not shown). These data confirmed that the blood particles represent protein aggregates derived from human blood.

In order to confirm the presence of these proteins within blood particles, we performed fluorescence microscopy using antibodies that react against HSA, HSF, or Apo-A1, followed by washing steps. Our observations indicated the presence of the three blood proteins in the particles (Fig. 6H–J). An antibody that recognizes heat-shock protein 70 (Hsp70), which was absent in the proteomics analysis of the particles (Supplementary Table 2), was used as a negative control (Fig. 6G). Co-localization of HSA and HSF was also observed within the particles (Fig. 6K). These results suggest that the refringent particles observed in human blood actually represent protein aggregates derived from blood.

### Prokaryotic 16S rDNA in human blood represents contamination

We examined whether the bacteria-like forms contained prokaryotic 16S rDNA using broad-range PCR analysis. Blood samples that were processed immediately after withdrawal (Fig. 7, lane 9) or incubated for one day (lane 10) or seven days (lane 11) produced 1.5-kb DNA products of low intensity. Positive controls using DNA template from *Esc﻿herichia* coli strain DH5α (Fig. 7, lane 1) or Stbl3 (lane 2), *Fusobacterium* nucleatum (lane 4), or aliquots of their culture medium (lane 3 for DH5α and lane 5 for *F. nucleatum*) produced 16S rDNA amplicons of low-to-high intensity. On the other hand, negative controls consisting of plain double-distilled water (Fig. 7, lane 7), double-distilled water processed for DNA extraction (lane 6), or DNA extraction buffer processed for DNA extraction (lane 8) also produced PCR products, suggesting the presence of low abundance contaminants in the reagents used.

The amplified PCR products were cloned and sequenced. Bacterial 16S rDNA genes corresponding to *Delftia acidovorans* and *Stenotrophomonas maltophilia* were identified in negative controls and incubated blood samples. *S. maltophilia* was identified earlier from the so-called pleomorphic bacteria isolated from human blood^18^. Notably, *Delftia* and *Stenotrophomonas* species represent common contaminants of PCR reagents^44, 45^, supporting our conclusion that the amplified DNA was the result of contamination.

## Discussion

The presence of pleomorphic bacteria in the blood of healthy humans would have important implications for disease transmission and blood transfusion. In the present study, we observed pleomorphic bacteria-like forms in human blood which were similar to the ones described in previous studies^3, 18^. These bacteria-like vesicles increased in number during incubation (Figs 1 and 3) and produced bacteria-like morphologies, including formations reminiscent of cells undergoing division (Fig. 2J and L). On the other hand, our observations indicate that these entities consist of lipid-delineated vesicles (Fig. 5B,D and Table 1) that harbor proteins commonly found in blood (Fig. 5E,F, and Supplementary Table 1). Of note, their increase in number during incubation *ex vivo* is consistent with a slow, time-dependent process of vesiculation and vesicle release from aging blood cells as observed through dark-field microscopy and TEM (Figs 2E,F and 4D).

RBCs have been found to spontaneously release vesicles during aging *in vitro* ^46^, a process attributed at least in part to decreased ATP production from glycolysis^47^. Blood cells also produce “myelinic forms,” long tubular protrusions of the cell membrane^46, 48^, similar to the structures observed here (Fig. 2F–H). It has been proposed that the release of such vesicles and myelinic forms may protect RBCs from premature engulfment by macrophages as vesicle release reduces the exposition of signals that induce phagocytosis, such as phosphatidylserine^49^. Besides, RBC-derived vesicles that are intravenously injected into laboratory animals are rapidly eliminated by macrophages via the scavenger receptor^50^, suggesting that the vesicles do not cause or transmit diseases under normal physiological conditions.

In addition to membrane-delineated vesicles, we observed vibrating refringent particles in human blood under dark-field microscopy (Fig. 1A–C, white arrows). These entities appear to be highly similar to earlier descriptions of mycrozymas, protits, or somatids made by previous authors^4^. On the other hand, we show that these vibrating particles consist of protein aggregates containing human proteins commonly found in blood (Fig. 6 and Supplementary Table 2), therefore confirming their non-living nature.

Our findings that the small refringent particles observed in human blood represent protein aggregates are consistent with the recent descriptions of “proteons” by Vodyanoy *et al*. who isolated metallic nanoclusters containing fragments of hemoglobin alpha-chain from blood^51, 52^. In addition to hemoglobin, our results indicate that various blood proteins including HSA, HSF and Apo-A1 may be incorporated within the protein particles (Fig. 6 and Supplementary Table 2). The refringent particles observed in blood are also similar to the mineralo-organic nanoparticles that we identified earlier in human blood and body fluids^26–42^—particles which we called bions^37^—although we did not examine the mineral or metallic composition of the particles in the present study. Our previous results suggest that membrane vesicles similar to the ones described here in human blood may serve as nucleators for the formation of mineralo-organic nanoparticles, which may aggregate and induce ectopic calcifications in the human body^31^. Further studies are needed to study the implications of protein particles, proteons and bions from physiological and pathophysiological points-of-view.

The results presented here indicate that LBA should not be used to monitor the presence of bacteria in human blood. Few studies have examined the reliability of LBA as a diagnostic tool in humans. A small prospective study that evaluated whether LBA could be used to diagnose cancer showed that only three out of 12 metastatic cancer patients could be identified by this technique, leading the authors to conclude that LBA is not suitable for this purpose^53^. Another preliminary study described a high level of variation in the interpretations made by LBA practitioners, and concluded that this technique should not be used as a diagnostic tool^54^.

Our results indicate that the bacteria-like forms observed in human blood by dark-field microscopy represent non-living membrane vesicles and protein particles that form during incubation of human blood. Based on these observations, we propose that previous literature on blood particles and pleomorphic bacteria needs to be reinterpreted from a different perspective.

## Methods

### Culture of bacteria-like forms

The use of human samples was approved by the Institutional Review Board of Chang Gung Memorial Hospital (Linkou, Taiwan; Document 1013415C) and the study was performed in accordance with the relevant guidelines. Written informed consents were obtained from the volunteers. Blood was collected from fasting healthy human volunteers (n = 30) by using a conventional venipuncture technique following sterilization of the skin with ethanol swabs. Whole blood was collected into Vacutainer tubes without anticoagulant (Becton, Dickinson & Company, Sparks, MD). In some experiments, blood samples were incubated at 30 °C for up to 1 week. All samples were manipulated under sterile conditions. Tubes were kept close during every experiment and liquid aliquots from clotted blood were withdrawn with a sterile syringe through the rubber liner. Absence of contaminating bacteria was confirmed by inoculating blood agar and brain heart infusion agar plates with the same aliquots, followed by incubation for at least 48 hrs at 37 °C.

Bacteria-like forms were isolated by centrifugating 10 ml of fresh or incubated blood at 800 × *g* for 15 min at 4 °C to pellet and remove cells and debris. The supernatant obtained this way was centrifuged again for 30 min at 10,000 × *g*. The resulting supernatant was centrifuged for 60 min at 15,000 × *g*. Material in supernatant was pelleted by ultracentrifugation at 200,000 × *g* for 2 hrs at 4 °C (Beckman Instruments, Pasadena, CA; rotor SW41). Pelleted bacteria-like forms were resuspended in HEPES buffer (20 mM HEPES, 140 mM NaCl, pH 7.4) and used for further analysis.

### Optical microscopy

Fresh or incubated human blood obtained from 30 healthy volunteers was deposited on a glass slide and observed without fixation or staining using a BX-51 optical microscope (Olympus, Tokyo, Japan) equipped with a 100× oil immersion objective with iris (UPlanFLN, Olympus) and a dark-field condenser (Cerbe Distribution, Sherbrooke, Canada). Each specimen was observed individually at a magnification of either 1,000 or 2,000× and images were acquired with a Spot Flex color camera (Diagnostic Instruments, Sterling Heights, MI). Two aliquots were observed from each specimen or treatment (incubation time).

### Dynamic light scattering

Particle sizing and counting was determined as before^30, 31^. Briefly, fresh blood (time 0) or blood incubated for 1 to 7 days was centrifuged at 800 × *g*, followed by filtration through 0.2 μm filter. Blood aliquots (0.8 ml) were submitted to DLS measurement using a Delta Nano Submicron Particle Analyzer (Beckman Coulter, Brea, CA). Mean particle sizes and numbers are shown as means ± standard deviation for experiments performed in triplicate (Fig. 3).

### Electron microscopy

For thin sections, the specimens were washed twice with HEPES buffer prior to dehydration with several washes of ethanol of gradually increasing concentration (10–90%). Samples were embedded with Epon 812 resin (Electron Microscopy Sciences, Fort Washington, PA). Thin sections of incubated blood or isolated bacteria-like forms were prepared as before^22^ using a Leica Ultracut UCT microtome (Leica Microsystems, Wetzlar, Germany). Specimens were examined under a JEM-100B (JEOL, Tokyo, Japan) TEM. For negative-staining, incubated blood or isolated bacteria-like forms were deposited onto formvar carbon-coated grids and negatively stained with 0.5% aqueous uranyl acetate, followed by drying overnight. Phosphatidylserine was detected as before^36^ using annexin V conjugated to 10-nm gold nanoparticles (BBI Solutions, Blaenavon, UK).

### Fluorescence microscopy

Ten ml of 7-day incubated human serum was centrifuged at 16,000 × *g* to remove cells and debris. Supernatant was treated with 0.1% (v/v) Triton X-100 detergent for 2 hrs to dissolve membrane vesicles or debris. The solution was centrifuged 30 min at 16,000 × *g* to remove membranes debris. Two hundred μl of supernatant was transferred onto a poly-lysine coated glass slide and allowed to dry. Two hundred and fifty μl of blocking solution (50 mM Tris, 150 mM NaCl, pH 7.6, 0.1% gelatin) was added for 1 hr at room temperature. Slides were washed three times with a modified blocking solution (solution above plus 0.05% Tween 20). Monoclonal antibody (anti-heat-shock protein 70, or α-Hsp 70, 1:50; sc-1060; Santa Cruz Biotechnology, Santa Cruz, CA) or polyclonal antibody (anti-human serum fetuin-A, or α-HSF, 1:4,000; anti-human serum albumin, or α-HSA, 1:4,000; or anti-human apolipoprotein A1, or α-h Apo-A1, 1:4,000; antibodies prepared in-house) was added for 1 hr at room temperature, followed by three washing steps. Goat anti-rabbit antibody coupled to fluorescein isothiocyanate (FITC; sc-2012; Santa Cruz Biotechnology) was added at 1:200 for 1 hr, followed by washing steps. Fluorescence was monitored using a Nikon Ti-E fluorescence microscope (Tokyo, Japan).

### Fluorescence spectroscopy

For DiR staining, bacteria-like forms corresponding to 0.5 μg of total proteins was mixed with 0.1 mM 1,1′-dioctadecyl-3,3,3′,3′-tetramethylindotricarbocyanine iodide (DiR; Molecular Probes, Carlsbad, CA) using a final volume of 1 ml. Bacteria-like forms were quantified by measuring total protein concentration using the Bradford protein assay (Bio-Rad, Hercules, CA). DiR samples were incubated 1 hr with continuous agitation. A negative control consisting of DiR added to HEPES buffer was processed in the same manner. THP-1 cells were purchased from the American Type Culture Collection (ATCC, Manassas, VA). Cells were cultured in RPMI 1640 cell culture medium containing 10% FBS and 100 U/ml of penicillin and streptomycin. 2 × 10^4^ THP-1 cells/ml were treated with DiR and used as positive control. Fluorescence was measured using a microplate reader (Spectra Max M5 Spectrophotometer, Molecular Devices, Sunnyvale, CA). Excitation was set at 748 nm and emission at 780 nm.

### Lipid analysis

Bacteria-like forms were isolated as described in the section “Culture of bacteria-like forms” from 3.5 ml of human blood. HEPES (200 μl) was added to resuspend the pellet obtained after ultracentrifugation. The concentration of phospholipids, cholesterol and triglycerides was quantified in resuspended bacteria-like forms (20 μl) by using commercial kits for phospholipids (BioAssay Systems), cholesterol, and triglycerides (BioVision).

### Western blotting

Proteins were resolved on 10% SDS-PAGE under denaturing and reducing conditions^24, 26^. Bacteria-like forms corresponding to 60 μg of proteins was dissolved in 5 × “loading buffer” (0.313 M Tris-HCl, pH 6.8, 10% SDS, 0.05% bromophenol blue, 50% glycerol, 12.5% β-mercaptoethanol) to obtain a final concentration of 1×. Protein mixtures were heated at 95 °C for 5 min. Polyclonal anti-HSA, HSF and Apo-A1 were prepared as before^36^. Primary antibodies consisted of goat polyclonal anti-tissue non-specific ALP (Santa Cruz Biotechnology; sc-15065), mouse monoclonal anti-Lamp2 (sc-18822), goat polyclonal anti-tumor necrosis factor receptor 1 (TNFR1; sc-31349), goat polyclonal anti-CD63 (sc-31214) and mouse monoclonal anti-RNA polymerase sigma S factor antibody (sc-101602). Secondary antibodies consisted of horse-radish peroxidase-conjugated anti-goat, anti-mouse, anti-sheep or anti-rabbit antibodies (Santa Cruz Biotechnology). Primary and secondary antibodies were diluted based on the instructions provided by the manufacturer. Blots were revealed using enhanced cheluminescence (GE Healthcare, Little Chalfont, UK). Membranes were stripped by using the ReBlot Western Blot Recycling Kit (Chemicon, Billerica, MA).

### PCR analysis

16S rDNA PCR was performed as before^18, 21^. Briefly, DNA from bacteria-like forms was isolated from 0.2 μm filtered blood (that had been incubated for one or seven days) using the commercial QIAamp DNA mini kit (Qiagen, Venlo, Netherlands). The primers used were: forward, 5′-AGAGTTTGATCCTGGCTCAG-3′; reverse, 5′-AAGGAGGTGATCCAGCCGCA-3′. Thirty-five cycles were performed as follows: denaturation for 1 min at 94 °C; annealing for 30 sec at 65 °C (or 70 °C); and extension for 90 sec at 72 °C. The GoTaq Hot Start Green Master Mix was used (Promega, Fitchburg, WI). Genomic DNA from *E. coli* (DH5α and Stbl3; Invitrogen, Carlsbad, CA) and *F. nucleatum* (kindly provided by Dr. David M. Ojcius) isolated as above or the bacteria’s culture medium was used as positive controls. Negative controls consisted of double-distilled water or the DNA extraction buffer processed as for extraction. Amplified PCR products were resolved on 1.5% agarose gel prior to staining with ethidium bromide. Amplicon size was estimated using the 100 Base Pair DNA ladder (Bayou Biolabs, Metairie, LA). DNA was purified using the Gel/PCR DNA Fragments Extraction kit (Geneaid, Taipei, Taiwan). DNA was cloned using the pGEM-T Vector Systems (Promega). Analysis of randomly picked clones was performed using standard T7 and SP6 primers. Sequencing was performed by Genomics (New Taipei City, Taiwan). Sequences were searched against the Nucleotide Blast database (National Center for Biotechnology Information, Bethesda, MD).

### Proteomics analysis

Proteomics analysis was performed as before^27, 30^. Briefly, bacteria-like forms and blood particles were isolated as described above, and washed twice with phosphate-buffered saline prior to processing for proteomics analysis. Samples were treated with dithiothreitol, iodoacetamide and trypsin as before^27, 30^. Dried peptides were mixed with formic acid (0.1%) and submitted to reverse-phase liquid chromatography (Zorbax 300SB-C18, Agilent Technologies, Santa Clara, CA). Peptide separation was performed using a 10-cm analytical C_18_ column (New Objective, Woburn, MA). Elution and analysis was done as before^27, 30^.

### Statistical analysis

Experiments were performed in triplicate. Values shown represent means ± standard error. Statistical comparison between control and experimental groups was performed using Student’s *t* test.

## Electronic supplementary material

Supplementary Information
