# Supplementary material for: Pleomorphic bacteria-like structures in human blood represent non-living membrane vesicles and protein particles
Source: Sci Rep. 2017 Sep 6;7:10650. doi: 10.1038/s41598-017-10479-8 (PMC5587737; doi:10.1038/s41598-017-10479-8)

Supplementary Information

Pleomorphic bacteria-like structures in human blood represent non-living membrane vesicles and protein particles

Jan Martel[1,2,3], Cheng-Yeu Wu[1,2,3,4], Pei-Rong Huang[1,2,5], Wei-Yun Cheng[1,2,3] and John D. Young[1,2,3,6,7]

[1]Center for Molecular and Clinical Immunology, Chang Gung University, Taoyuan, Taiwan.

[2]Laboratory of Nanomaterials, Chang Gung University, Taoyuan, Taiwan.

[3]Chang Gung Immunology Consortium, Linkou Chang Gung Memorial Hospital, Taoyuan, Taiwan.

[4]Research Center of Bacterial Pathogenesis, Chang Gung University, Taoyuan, Taiwan.

[5]Department of Molecular and Cellular Biology, College of Medicine, Chang Gung University, Taoyuan, Taiwan.

[6]Laboratory of Cellular Physiology and Immunology, Rockefeller University, New York, NY.

[7]Biochemical Engineering Research Center, Ming Chi University of Technology, Taipei, Taiwan.

Supplementary Information

Supplementary Table 1. Proteins identified in membrane vesicles derived from blood

No.	Protein Identified	UniProt Accession No.	MW (kDa)	Membrane Vesicles	
				Spectra	emPAI
1	Complement C3	P01024	187.0	544.5	15.2
2	Complement C4-B	P0C0L5	192.6	135.4	6.3
3	Complement C4-A	P0C0L4	192.7	129.6	6.1
4	Alpha-2-macroglobulin	P01023	163.2	89.3	4.6
5	Complement C5	P01031	188.2	50.0	2.1
6	Complement factor H	P08603	139.0	44.6	2.6
7	Ceruloplasmin	P00450	122.1	42.2	2.6
8	C4b-binding protein alpha chain	P04003	67.0	35.9	2.8
9	Complement C1r subcomponent	P00736	80.1	33.5	3.1
10	Inter-alpha-trypsin inhibitor heavy chain H4	Q14624	103.3	33.5	2.3
11	Complement factor B	P00751	85.5	33.0	2.9
12	Complement C1s subcomponent	P09871	76.6	31.5	2.5
13	Plasminogen	P00747	90.5	28.6	2.7
14	Vitamin D-binding protein	P02774	52.9	28.1	5.9
15	Serotransferrin	P02787	77.0	28.1	4.0
16	Inter-alpha-trypsin inhibitor heavy chain H1	P19827	101.3	24.3	1.5
17	Hemopexin	P02790	51.6	23.8	2.9
18	Human serum albumin	P02768	69.3	23.3	3.7

19	Histidine-rich glycoprotein	P04196	59.5	16.0	1.0
20	Complement factor I	P05156	65.7	15.5	1.5
21	Attractin	O75882	158.4	14.6	0.6
22	Beta-2-glycoprotein 1	P02749	38.3	14.6	2.2
23	Complement C2	P06681	83.2	14.6	1.7
24	Vitamin K-dependent protein S	P07225	75.1	14.6	1.2
25	Ig mu chain C region	P01871	49.3	14.1	2.0
26	Gelsolin	P06396	85.6	14.1	1.1
27	Inter-alpha-trypsin inhibitor heavy chain H2	P19823	106.4	14.1	0.5
28	Complement component C6	P13671	104.7	13.6	1.1
29	Afamin	P43652	69.0	13.1	1.4
30	Kininogen-1	P01042	71.9	12.6	1.5
31	Ig gamma-1 chain C region	P01857	36.1	12.1	1.8
32	Protein AMBP	P02760	39.0	12.1	2.2
33	Pigment epithelium-derived factor	P36955	46.3	12.1	1.7
34	Ig gamma-3 chain C region	P01860	41.3	11.2	1.2
35	Complement component C8 alpha chain	P07357	65.1	10.7	1.3
36	Ficolin-3	O75636	32.9	10.2	2.1
37	Fibrinogen beta chain	P02675	55.9	10.2	1.5
38	Retinol-binding protein 4	P02753	23.0	10.2	4.0
39	Alpha-1B-glycoprotein	P04217	54.2	10.2	1.9
40	Complement component C8 gamma chain	P07360	22.3	10.2	3.4
41	Complement component C7	P10643	93.5	10.2	1.0

42	Angiotensinogen	P01019	53.1	9.2	0.9
43	Ig delta chain C region	P01880	42.2	9.2	1.2
44	Vitronectin	P04004	54.3	9.2	1.0
45	Zinc-alpha-2-glycoprotein	P25311	34.2	9.2	2.0
46	Haptoglobin	P00738	45.2	8.7	2.0
47	Fibrinogen gamma chain	P02679	51.5	8.3	0.9
48	Human serum fetuin-A	P02765	39.3	8.3	1.1
49	C4b-binding protein beta chain	P20851	28.3	8.3	1.7
50	Properdin	P27918	51.2	8.3	0.9
51	Ig alpha-1 chain C region	P01876	37.6	7.3	1.4
52	Clusterin	P10909	52.5	7.3	0.9
53	Galectin-3-binding protein	Q08380	65.3	7.3	1.0
54	Serum amyloid P-component	P02743	25.4	6.8	2.2
55	Mannan-binding lectin serine protease 1	P48740	79.2	6.8	0.5
56	Ig gamma-2 chain C region	P01859	35.9	6.3	1.2
57	Fibrinogen alpha chain	P02671	94.9	6.3	0.4
58	Cholinesterase	P06276	68.4	6.3	0.7
59	Plasma kallikrein	P03952	71.3	5.8	0.8
60	Thyroxine-binding globulin	P05543	46.3	5.8	1.1
61	Carboxypeptidase N subunit 2	P22792	60.5	5.8	0.7
62	Prothrombin	P00734	70.0	5.3	0.4
63	Ig lambda-2 chain C regions	P0CG05	11.3	5.3	5.0
64	N-acetylmuramoyl-L-alanine amidase	Q96PD5	62.2	5.3	0.5

65	CD5 antigen-like	O43866	38.1	4.9	0.9
66	Antithrombin-III	P01008	52.6	4.9	0.5
67	Ig kappa chain C region	P01834	11.6	4.9	3.0
68	Complement C1q subcomponent subunit B	P02746	26.7	4.9	0.9
69	Plasma protease C1 inhibitor	P05155	55.1	4.9	0.4
70	Complement factor H-related protein 1	Q03591	37.6	4.9	0.9
71	Apolipoprotein A-I	P02647	30.8	4.4	1.0
72	Transthyretin	P02766	15.9	4.4	2.7
73	von Willebrand factor	P04275	309.1	4.4	0.1
74	Corticosteroid-binding globulin	P08185	45.1	4.4	0.9
75	Hemoglobin subunit beta	P68871	16.0	4.4	3.7
76	Heparin cofactor 2	P05546	57.0	3.9	0.5
77	Inter-alpha-trypsin inhibitor heavy chain H3	Q06033	99.8	3.9	0.3
78	Complement C1q subcomponent subunit C	P02747	25.8	3.4	0.6
79	Complement component C9	P02748	63.1	3.4	0.3
80	Dopamine beta-hydroxylase	P09172	69.0	3.4	0.4
81	Coagulation factor XII	P00748	67.7	2.9	0.4
82	Alpha-1-antitrypsin	P01009	46.7	2.9	0.6
83	Alpha-1-antichymotrypsin	P01011	47.6	2.9	0.6
84	Complement C1q subcomponent subunit A	P02745	26.0	2.9	0.9
85	Leucine-rich alpha-2-glycoprotein	P02750	38.2	2.9	0.6
86	Alpha-1-acid glycoprotein 1	P02763	23.5	2.9	0.7
87	Tetranectin	P05452	22.5	2.9	0.7

88	Complement component C8 beta chain	P07358	67.0	2.9	0.5
89	Biotinidase	P43251	61.1	2.9	0.4
90	Alpha-2-antiplasmin	P08697	54.5	2.4	0.5
91	Ficolin-2	Q15485	34.0	2.4	0.4
92	Ig kappa chain V-III region SIE	P01620	11.8	1.9	0.4
93	Fibulin-1	P23142	77.2	1.9	0.2
94	Actin, cytoplasmic 1	P60709	41.7	1.9	0.4
95	Ig kappa chain V-I region EU	P01598	11.8	1.5	1.0
96	Ig heavy chain V-III region BRO	P01766	13.2	1.5	0.9
97	Apolipoprotein A-II	P02652	11.2	1.5	1.1
98	Fibronectin	P02751	262.5	1.5	0.0
99	Catalase	P04040	59.7	1.5	0.3
100	Thrombospondin-1	P07996	129.3	1.5	0.1
101	Mannose-binding protein C	P11226	26.1	1.5	0.4
102	Extracellular matrix protein 1	Q16610	60.6	1.5	0.2
103	Coagulation factor XI	P03951	70.1	1.0	0.1
104	Lipopolysaccharide-binding protein	P18428	53.3	1.0	0.2
105	Hepatocyte growth factor-like protein	P26927	80.3	1.0	0.2
106	IGF-binding protein complex acid labile subunit	P35858	66.0	1.0	0.1

Proteins were ranked based on the sum of spectra of the two samples.

Supplementary Table 2. Proteins identified in refringent particles isolated from blood

No.	Protein Identified	UniProt Accession No.	MW (kDa)	Particles		Autoclaved Particles	
				Spectra	emPAI	Spectra	emPAI
1	Hemoglobin subunit beta	P68871	16.0	542.8	623.5	734.5	16.0
2	Hemoglobin subunit alpha	P69905	15.2	377.1	128.5	470.7	7.7
3	Hemoglobin subunit delta	P02042	16.0	280.8	58.9	222.4	11.9
4	Serum albumin	P02768	69.3	–	–	269.0	1.6
5	Complement C3	P01024	187.0	88.1	1.6	77.6	0.2
6	Carbonic anhydrase 1	P00915	28.9	101.0	12.6	–	–
7	Hemoglobin subunit gamma-1	P69891	16.1	68.1	20.5	–	–
8	Peroxiredoxin-2	P32119	21.9	29.4	7.0	25.9	0.5
9	Carbonic anhydrase 2	P00918	29.2	23.5	3.2	31.0	0.3
10	Ceruloplasmin	P00450	122.1	36.4	1.0	–	–
11	Ig gamma-1 chain C region	P01857	36.1	–	–	36.2	0.4
12	Catalase	P04040	59.7	31.7	2.1	–	–
13	Apolipoprotein B-100	P04114	515.3	22.3	0.1	–	–
14	Apolipoprotein A-I	P02647	30.8	20.0	2.9	–	–
15	L-lactate dehydrogenase B chain	P07195	36.6	9.4	0.6	10.3	0.3
16	Antithrombin-III	P01008	52.6	2.3	0.1	15.5	0.2
17	Beta-2-glycoprotein 1	P02749	38.3	7.0	0.9	10.3	0.2
18	Hemopexin	P02790	51.6	16.4	0.8	–	–
19	Flavin reductase (NADPH)	P30043	22.1	16.4	2.7	–	–

20	Complement C4-A	P0C0L4	192.7	12.9	0.2	–	–
21	Purine nucleoside phosphorylase	P00491	32.1	11.7	0.9	–	–
22	Fetuin-A	P02765	39.3	11.7	0.5	–	–
23	Selenium-binding protein 1	Q13228	52.4	11.7	0.8	–	–
24	Superoxide dismutase [Cu-Zn]	P00441	15.9	10.6	1.8	–	–
25	Vitamin D-binding protein	P02774	52.9	10.6	0.5	–	–
26	Vitronectin	P04004	54.3	10.6	0.6	–	–
27	Inter-alpha-trypsin inhibitor heavy chain H4	Q14624	103.3	10.6	0.3	–	–
28	Apolipoprotein A-IV	P06727	45.4	9.4	0.5	–	–
29	Inter-alpha-trypsin inhibitor heavy chain H1	P19827	101.3	9.4	0.3	–	–
30	Complement factor B	P00751	85.5	8.2	0.3	–	–
31	Transthyretin	P02766	15.9	8.2	1.8	–	–
32	Alpha-1B-glycoprotein	P04217	54.2	8.2	0.5	–	–
33	Inter-alpha-trypsin inhibitor heavy chain H2	P19823	106.4	8.2	0.5	–	–
34	14-3-3 protein beta/alpha	P31946	28.1	8.2	0.6	–	–
35	Phosphoglycerate kinase 1	P00558	44.6	7.0	0.9	–	–
36	Angiotensinogen	P01019	53.1	7.0	0.3	–	–
37	Histidine-rich glycoprotein	P04196	59.5	7.0	0.3	–	–
38	Triosephosphate isomerase	P60174	30.8	7.0	0.7	–	–
39	Apolipoprotein E	P02649	36.1	5.9	0.4	–	–
40	Glyceraldehyde-3-phosphate dehydrogenase	P04406	36.0	5.9	0.4	–	–
41	Calpastatin	P20810	76.5	5.9	0.2	–	–
42	Hypoxanthine-guanine phosphoribosyl transferase	P00492	24.6	4.7	0.7	–	–

43	Apolipoprotein A-II	P02652	11.2	4.7	2.0	–	–
44	Plasma protease C1 inhibitor	P05155	55.1	4.7	0.2	–	–
45	Transitional endoplasmic reticulum ATPase	P55072	89.3	4.7	0.1	–	–
46	Peptidyl-prolyl cis-trans isomerase A	P62937	18.0	4.7	1.0	–	–
47	Glutathione S-transferase omega-1	P78417	27.5	4.7	0.6	–	–
48	Retinal dehydrogenase 1	P00352	54.8	3.5	0.2	–	–
49	Prothrombin	P00734	70.0	3.5	0.2	–	–
50	Alpha-1-antichymotrypsin	P01011	47.6	3.5	0.4	–	–
51	Protein AMBP	P02760	39.0	3.5	0.2	–	–
52	Gelsolin	P06396	85.6	3.5	0.2	–	–
53	Heat shock cognate 71 kDa protein	P11142	70.9	3.5	0.1	–	–
54	Delta-aminolevulinic acid dehydratase	P13716	36.3	3.5	0.6	–	–
55	Acylamino-acid-releasing enzyme	P13798	81.2	3.5	0.2	–	–
56	Alpha-hemoglobin-stabilizing protein	Q9NZD4	11.8	3.5	1.0	–	–
57	Coagulation factor X	P00742	54.7	2.3	0.2	–	–
58	Complement C5	P01031	188.2	2.3	0.1	–	–
59	Protein S100-A6	P06703	10.2	2.3	1.2	–	–
60	Proteasome subunit alpha type-2	P25787	25.9	2.3	0.4	–	–
61	Serine/threonine-protein phosphatase 2A activator	Q15257	40.6	2.3	0.2	–	–

Proteins were ranked based on the sum of spectra of the two samples.

Supplementary Figure 1. Full, uncropped Western blots of Figure 5E. See the legend of Figure 5 for details.

Figure 5E, Lane 1

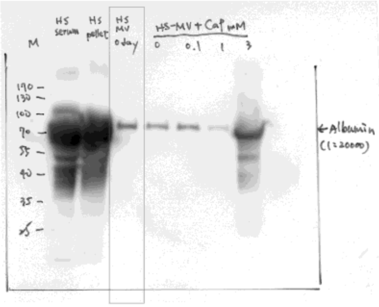

Figure 5E, Lane 2

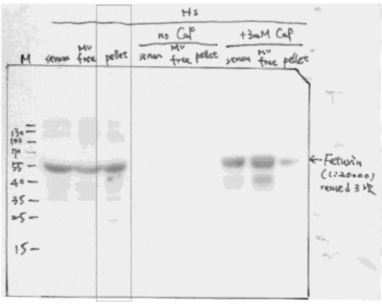

Figure 5E, Lane 3

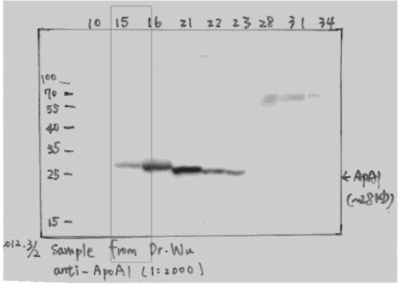

Figure 5E, Lane 4

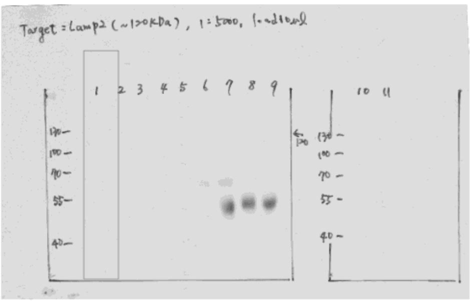

Figure 5E, Lane 5

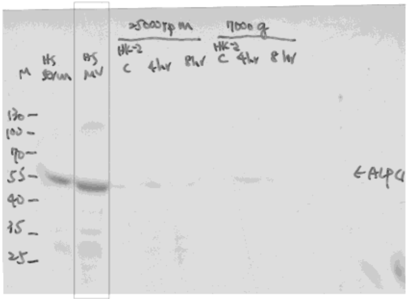

Figure 5E, Lane 6

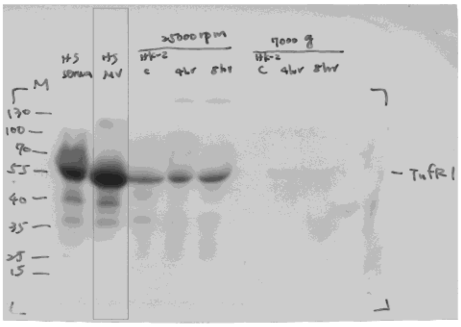

Figure 5E, Lane 7

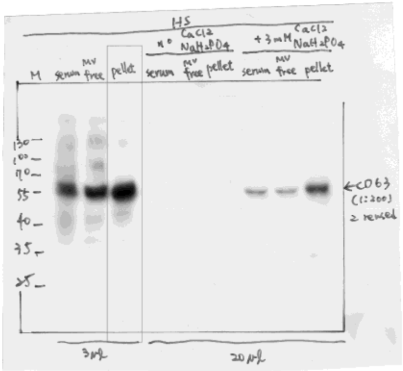

Figure 5E, Lane 8

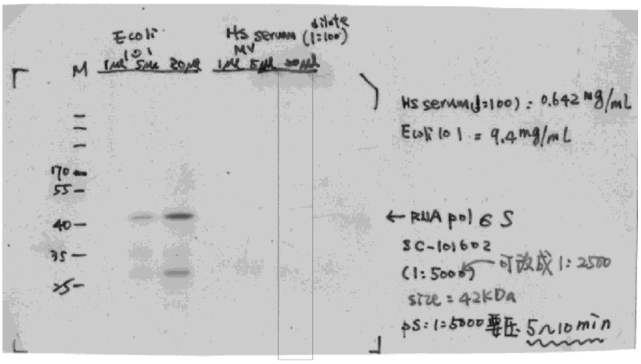

Supplement: Supplementary file 1 — Supplementary Information [file 41598_2017_10479_MOESM1_ESM.pdf]
